# Achieving sustainable development goals through digitalising creative works: some evidence from social enterprises in Indonesia

**DOI:** 10.1007/s44265-023-00011-4

**Published:** 2023-06-15

**Authors:** Aluisius Hery Pratono, Catharina Badra Nawangpalupi, Ari Sutanti

**Affiliations:** 1 Faculty of Business and Economics, Universitas Surabaya, Raya Kalirungkut, Surabaya, 60293 Indonesia; 2 Faculty of Engingeering, Universitas Katholik Parahyangan, Jl. Ciumbuleuit No.94, Cidadap, Kota Bandung, 40141 Indonesia; 3 British Council Indonesia, Jl. Prof. DR. Satrio No.3/5, RT.18/RW.4, Kuningan, Setiabudi, Jakarta Selatan, 12940 Indonesia

**Keywords:** Sustainable development goals, Social entrepreneurship, Stewardship theory, Cultural identity

## Abstract

This article aims to examine how businesses support the SDGs by exploring the role of social enterprises in supporting creative workers by adopting digital economic activities. This study adopts the inductive qualitative approach by observing the creative industry, conducting focus group discussions, and interviewing the main stakeholders to arrive at four findings. The findings indicate that social enterprises (1) encourage the creative workers to embrace their cultural identities by providing a digital platform, (2) support the creative workers to deal with customers by exploiting resourceful social networks, (3) promote inclusive community governance by adopting the crowdfunding platform, and (4) help the creative workers gain support from donors by showing their excellent pilot projects. The article extends the debate between neoclassical economics with profit-oriented goals and social enterprise approaches by highlighting the role of stakeholder, triple bottom line, and identity theories.

## Introduction

The digital economy gains acknowledgement as one of the most critical elements for business entities in achieving sustainable growth. The United Nations General Assembly declares that 2021 is the international year of the creative economy for sustainable development (UNTAC 2019). Many creative enterprises work with young people experiencing marginalisation, while others work through a culture of peace and non-violence, essential for sustainable development (Zheng et al. 2021). Hence, sustainability involves the business model of social enterprises (SEs) that stipulate specific entrepreneurial capacities to generate revenue (McQuilten et al. 2020).

It is no coincidence that social enterprises provide digital resources to support democracy, equality, and human dignity, equality, and democracy, which are essential elements in the achievement of sustainable development goals (UNTAC 2019). The British Council (2020) indicates that 48 per cent of the creative enterprises in Indonesia adopt a social enterprise model with creative products, such as craft, fashion, culinary, and eco-tourism, that generate jobs faster than other businesses for young, women, and disabled people. Hence, the United Nations call for effective partnership in supporting partnership toward the 2030 Agenda for Sustainable Development (Stibbe and Prescott 2020).

In Indonesia, social enterprises attempt to gain support from digital platforms to increase product and service diversification by allowing creative workers to integrate income-generating and social activities (Pratono et al. 2019). However, there needs to be more evidence with in-depth case study work about why and how SEs build commitment to the SDGs (Littlewood and Holt 2018). Understanding the business motivations to attain specific SDGs is essential to understand the conditions necessary for achieving the 2030 Agenda, where companies vary in size, cultural condition, legislative framework, and type of capitalism (Mio et al. 2020).

Studies need to explore additional enablers, which become catalysts to promote social innovation and undermine the innovation process following the contradiction in the measure of social innovation with credible solutions (Mihci 2020). The COVID has devastated the projects following the lockdown policies. In April 2020, the United Nations released a policy framework to support social and economy, such as protecting health systems, helping people cope with adversity, supporting small and medium enterprises, guiding fiscal stimulus for the most vulnerable, and promoting social cohesion (United Nations 2021).

This study seeks to understand how businesses support the SDGs by exploring the role of social enterprises in supporting creative workers by adopting digital economic activities. This study adopts the inductive qualitative approach by observing the creative industry, conducting focus group discussions, and interviewing the main stakeholders to arrive at four findings. Hence, we derive the main research question into four mini-research questions: (1) how social enterprises help the creative workers deal with their unique identity, (2) how social enterprises help the creative workers meet the customers’ expectation, (3) how social enterprises support inclusiveness, and (4) how social enterprises gain support from donors. The article provides insightful findings to the stewardship and stakeholder theories to understand how the creative enterprise achieves the SDGs.

## Literature

### Social enterprise

The social enterprise gained acknowledgement from Nobel committees following the seminal work of Muhammad Yunus on social microfinance for poverty reduction in 2006 (Haugh 2012). Social enterprise refers to an organisation that continuously produces and sells goods and services to benefit the community by raising concerns about autonomy and risk-taking behaviour (Nyssens 2006). On the contrary, neo-classical theory traditionally presumes that firms seek to maximise profits by producing goods and services in high demand and at a higher price than the cost of production (Henry 2012). The concept raises disputes between mainstream theories.

First, from a micro perspective, the agency theory believes incentive structures and contracts can explain the managers’ and employees’ behaviour (Jensen and Meckling 1976). In this theory, managers are seen as owners’ agents and are motivated to act in the owners’ best interest, which is typically to maximise profits. The theory of the firm is an economic concept that attempts to explain how firms operate and make decisions. It seeks to answer questions such as why firms exist and what factors determine their behaviour and performance. The firm’s primary goal is to maximise profits by producing goods and services for a higher price than the cost of production.

Secondly, shareholder theory holds that the primary goal of a firm is to maximise shareholder value. According to this theory, firms should focus on generating profits and maximising the value of their shares to benefit shareholders. Agency theory and shareholder theory are both economic theories that focus on firms’ behaviour and decision-making. However, there are critical differences between the two theories. Agency theory focuses on the relationship between the principal (the owners or shareholders of the firm) and the agent (the managers and employees who work for the firm). It seeks to explain how this relationship affects the agent’s behaviour and the firm’s overall performance. In contrast, shareholder theory focuses on maximising shareholder value as the firm’s primary goal.

At the macro level, stakeholder theory argues that businesses should consider the interests of all stakeholders, including customers, employees, suppliers, and the community, not just shareholders. Advocates of this theory believe that by creating value for all stakeholders, the firm can achieve long-term success and sustainability. The social mission comes from the initiative to respond to social problems, such as unemployment during the crisis (Pratono et al. 2019). Adopting social enterprise is about serving a higher purpose in addition to the necessity of delivering profits (Freeman et al. 2020).

While stakeholder theory has gained popularity recently, some critics argue that it may only be relevant in some conditions. This is because the stakeholders vary from the employees to the end customers who seek to co-create value through relationships (Barney and Harrison 2020). For example, when a firm is facing financial distress, stakeholders may have to make sacrifices to ensure the survival of the firm. In such situations, the theory may not be relevant as the firm must prioritise its shareholders’ interests to survive. Furthermore, in some cases, the institutional context encourages firms to prioritise specific stakeholders over others. For example, labour laws may require firms to prioritise the interests of their employees over those of their shareholders. Hence, business leaders fabricate a strong sense of identity by exploiting multiple categorisations that may increase identity power (MacClacy 2020).

Thirdly, the triple bottom theory argues that businesses should aim to maximise not just profits but also social and environmental benefits. The triple bottom line refers to three areas of concern: economic, social, and ecological. Creative enterprises present a business model that incorporates a component of social responsibility by shaping personal growth and entrepreneurial identity (Pearse and Peterlin 2019). Hence, the environment that supports the social mission continuously evolves with great effort to balance social-business purposes, including goodwill factors (Tsai et al. 2020), stakeholders’ pressure (Pratono and Han 2021), and technological turbulence (Sewell et al. 2021).

There may be some situations where the triple bottom line theory is less relevant. For example, when a company is facing a short-term financial crisis, its immediate priority may be to restore financial stability, even if it means sacrificing social or environmental goals in the short term. On the other hand, start-up companies may be more focused on achieving financial stability and growth in the early stages of their development. As a result, they may need more resources to devote to social and environmental initiatives. In such cases, the TBL theory may be less relevant as the company may need to prioritise financial performance to establish itself.

Last, identity theory seeks to understand various identities that involve negotiation interaction by examining the role of performance, self-concept, and social structure (Stets and Serpe 2013). Finally, stewardship theory seeks to understand how a social enterprise seeks the effectiveness of financial resources within the organisation (Low 2006). The social enterprise demonstrates how the social mission intertwines with economic activities, which could be mission-centric or mission-related (Defourny et al. 2020). Arts engagement provides an experience of extraordinary mental well-being (Davies et al. 2015). Hence, the identity dimensions emanate from the role of cultural leaders who seek to create a sense of belonging (Sewell et al. 2021).

Cultural identities determine community behaviour by allowing individuals to adopt values from a more extensive set of cultural beliefs. Hence, the power of cosmopolitanism through modern information technology leads individuals to adopt global culture (Friedman 1994). As a result, globalisation drives some cultural identity gets lost while others gain better acknowledgement (Jessen et al. 2011). Moreover, protecting cultural identity induces ethnocentric behaviour and poses a bid for power (Pratono and Arli 2020). Social media contributes to the creative works in finding new identity by reflecting on the social issue to find a solution that results in mutual benefits, providing social support and connection (Fujita et al. 2018).

### The context

The definition of creative enterprises is different from one context to another. In Indonesia, the Government of Indonesia poses Law No 24/2019, which states that a creative economy presents added value from intellectual property that springs from local culture and technology. The government consider 17 subsectors, including the food industry, which plays a pivotal role in Indonesia’s informal sectors. The definition is different from other countries. In the United Kingdom, the Department of Culture, Media and Sports presents thirteen business sectors to the creative industries, which exclude food as creative work.

The Government of Indonesia also formed the Ministry of Tourism and Creative Industry in 2015 to support the creative industry in this country. In addition, many other donors attempt to help social and creative enterprises. British Council, ASEAN Foundation, GIZ, and Asian Development Bank are among those that provide a resource to promote social and creative enterprise. However, there is no legal form to acknowledge the social enterprise model.

In the past few years, Indonesian start-up businesses have been emerging following the number of creative workers and information technological progress. However, many early-stage firms suffer and seek survivability, while others are desperate for expansion. Therefore, they also needed help to gain support from angel investors and other venture capital companies. On the other hand, a few venture capital companies prefer to adopt pre-seed funding, which gets the operation off the ground or seed funding, which helps a firm to finance product development and market research due to the high level of risk.

The Indonesian Financial Services Authority (OJK) poses Regulation No. 37/2018, which attempts to support start-up firms with various alternative financial resources. Since this mechanism allows the private company to offer and sell shares in a way that is like an initial public offering (IPO), the OJK attempts to control the crowdfunding practice and ensure that the consummation of crowdfunding will not alter its objective, primarily through the criteria for providers, investors, and issuers (the three main parties involved) and disclosure requirements.

## Research method

### Research design

This study adopts an interpretative approach to understand how creative workers discover the situation of achieving the 2030 Agenda by adopting the social enterprise model. This approach is relevant to develop theories by examining the meanings and interpretations (Ezzy 2002). This study focuses on meanings and interpretations of the concept of social enterprise by elaborating the stewardship and stakeholder theory to provide a more helpful understanding of the phenomenon. This approach addresses research questions based on participants’ experiences and perspectives (Hammarberg et al. 2016).

This study attempts to analyse how participants perceive and make sense of things happening to them. Approaching the data by focusing on the research purposes allows the authors to extend the main research question derived into four sub-questions: (1) how the social enterprises support creative workers to generate more creative products, (2) sell their products, (3) empower the creative community, and (4) gain support from the stakeholders. Hence, this study conducted a qualitative approach with a series of focus group discussions, interviews, and observations to address the research purposes. The research participants are the creative workers, business leaders, donors, and investors concerned with the Indonesian creative enterprise industry.

### Data collection

The idea for this study comes from the Indonesia Development Forum 2017, in which the authors were invited to present the preliminary investigation of social enterprise. This Forum motivated the authors to discuss the role of social enterprise in achieving Sustainable Development Goals. The research work then continued into the Indonesian Development Forum 2019, which challenged the authors to explore the contribution of creative enterprise in supporting inclusive economic growth. The authors also benefited from extending the networks from the Forum, which then allowed the authors to meet various stakeholders in Agenda 2030, especially the creative communities in multiple cities in Indonesia, the policymakers, and the international development agencies.

The next step was the first round of focus group discussions (FGDs), which started in three major cities: Jakarta, Bandung, and Surabaya. This first round concerns gaining support from the main stakeholders. Hence, the discussion concerns the purposes of the study and research questions, which spring from the research theme of Sustainable Development Goals—initially, four research questions spring from the discussion.

In the second round of focus group discussion, the authors conducted online meetings with the previous sessions’ research participants to ensure that the results met their expectations. The research participant for the first and second rounds came from the policymakers, international donors, business communities, and creative communities. Hence, the discussion narrows down the main questions into two sub-research questions.

The next round came to a series of focus group discussions that concerned creative communities in 12 cities from Medan to Ambon following the research participants’ support at the previous meetings. As a result, the creative workers in 12 cities agreed to support this study. However, after accomplished discussions in eight cities, the researchers could not conduct offline focus discussions following COVID-19. Hence, the researcher must amend the original proposal for online data collection.

This study considers the anonymity of the participants to avoid risks and to assure data credibility (Ibbett and Brittain 2019). The authors selected the invited participants through online discussions and interviews to prevent unexpected intruders. An additional issue arises in a focus group discussion following the possibility that the participants compromise the confidentiality of the data (Lobe et al. 2020). To avoid the risk, the introduction for the focus group discussions began with the statement: “Participants should be aware that there is no guaranty of confidentiality in a group setting.”

### Data analysis

The interpretative approach involves coding analysis following elaborating on the phenomenon in question. The interpretative approach allows the authors to answer the research questions by examining the initial analytic thoughts toward the significant knowledge of the data. The researchers adopt both inductive and deductive approaches to develop the codes. The inductive approach involves a coding process using phrases or terms from the research participants. Adopting the deductive approach helps the analysis deal with the risk of unfocused analysis. At the same time, the inductive method must have many codes to provide high-level categories, which allows the study to support the findings (Linnenberg and Korsgaard 2019; Nowell et al. 2017).

The authors generated initial categories during the data collection by focusing on the research questions. The initial analysis concerned the gap between collected data and research questions that aligns with understanding previous theories (Grodal et al. 2021). Furthermore, inviting research participants to share their experiences offers a learning process beyond the initial research topic (Hammarberg et al. 2016). Hence, the authors categorised the initial findings by reconstructing the research purposes and questions.

The first step highlights the research questions involving funding and expert focus group discussion. We present the research gap after conducting a comprehensive review of relevant literature, such as published research papers, journal articles, books, and conference proceedings, with the current state of knowledge and identify the key topics from social and creative perspectives. The funding agency shares their expectation, while the experts highlight some keywords by sharing their experiences.

The second step begins with open coding by identifying key concepts, themes, or patterns from the first round of focus discussions. The researchers invited the participants to label their keywords during the discussions. We also identified instances where participants express ideas or concepts in their language and assign them as codes. This approach helps maintain the participants’ perspectives and ensures the codes present their voices. Finally, we compared the findings from the focus group discussions with data from interviews and observation.

Thirdly, the researchers organise the open codes into categories or themes to generate axial codes. We looked for commonalities, patterns, or relationships between codes and grouped them accordingly. This process creates a coding framework or axial codes that outlines the categories and their definitions. We compared the concepts that emerged from the literature. We also reviewed the recordings of the focus group discussions to understand the topics, ideas, and themes discussed comprehensively. Hence, we invited the expert groups to review the coding process.

Last, the researchers continuously revise and refine the codes to gain a deeper understanding of the data. We merge similar codes, split codes when necessary, or create new codes to capture nuanced meanings. We also develop summaries or memos for each code, briefly describing the content or meaning associated with the code. In addition, we conducted expert focus group discussions to look for insights, variations, or contradictions within and across the focus group discussions. We also develop interpretations, draw conclusions, and support them with evidence from the coded data.

### Profile of research participants

This study divided the research participants into three groups. The first group is the ones who participated in the first-round focus discussion groups in Jakarta and Bandung. They represented international donors, investors, and business leaders concerned with a social enterprise model. Some of those business leaders served as professional architects who established creative communities with 200–500 members from creative workers.

The second group represented local business leaders from different cities, where we conducted focus group discussions. During the meeting, one or two business leaders had a solid creative business background in those cities. They also managed other business activities, such as cafés, museums, galleries, and other public spaces that provided activities for local creative workers in the towns. Those business leaders have been active in the creative industry for over 20 years and focused on the local markets with 25–50 full-time employees. Those leaders also knew each other and had annual meetings to promote the creative industry at national levels.

Another group of research participants represented young creative people who managed a small-scale business with two or three staff. The young creative people aged 18 to 25 operated one innovative firm mostly at local markets. However, we found two to five artists in each city that served some international customers. They were eager to transform their creative work into an online business by actively joining a weekly meeting the business leaders organised. Some of them attempted to establish an online gallery with support from the government or donors.

The third-round discussion gained support from163 research participants represented creative workers from 12 cities. They worked in various business activities, most concerned with food and fashion, which are pivotal in Indonesia’s economy. That may be why the Ministry of Tourism and Creative Industry should involve those subsectors in creative works. In addition, the research participants stated that their careers are related to some of the thematic areas in SDGs, from decent jobs to sustainable cities (Tables 1 and 2).

**Table 1:** Key theoretical concepts

**Key theories**	**Key words**
Social enterprise (Nyssens 2006; Defourny et al. 2020)	Social purpose
	Economic activities
	Participatory governance
Stewardship theory (Low 2006; Friedman 1994)	Long-term contractual relationship
	Collective goal and involvement
	Trust and reputation
Stakeholder theory (Fujita et al 2018; Barney and Harrison 2020)	Internal stakeholder
	External stakeholder
	Value creation

**Table 2:** Profile of focus group discussions

**No**	**Area**	**Number of participants**	**Core business activities**	**Thematic area**
1	Jakarta	14	Fashion, food, housing	Decent work, education, gender equality, hunger, housing, climate change, energy
2	Bandung	16	Fashion, food, art graphic design	Poverty, decent work, education, gender equality, sustainable city
3	Surabaya	15	Fashion, food, housing	Poverty, hunger, gender equality, housing, sustainable city
4	Malang	16	Fashion, food, art graphic design, and dancing	Decent work, poverty, education, climate change
5	Denpasar	15	Fashion, food, museum, and recycled content products	Decent work, economic gap, poverty, sustainable city, clean water and sanitation
6	Yogyakarta	13	Fashion, traditional dancing, art	Decent work, education, climate change
7	Makassar	16	Fashion, movie, museum	Decent work, education, climate change, good health
8	Pontianak	15	Fashion, movie, food	Poverty, decent work, education, climate change, good health
9	Medan^a^	14	Fashion, food, art graphic design	Decent work, education, gender equality, sustainable city
10	Ambon^a^	9	Fashion, food, music	Poverty, decent work, education, climate change, life below water

^a^Online discussion

Table 3 shows the participants’ acknowledgement of their development initiatives aligned with the SDGs. The participants that work in the fashion and food industry claim that their organisations provide employment and income opportunities by generating jobs, paying fair wages, and providing safe working conditions. The initiatives contribute to poverty reduction and economic empowerment. Additionally, promoting body positivity and diversity in fashion can improve mental and emotional well-being. Collaborating with non-profit organisations and government agencies to implement effective food aid programs and social safety nets encourages the industry to adopt a social enterprise model.

**Table 3:**
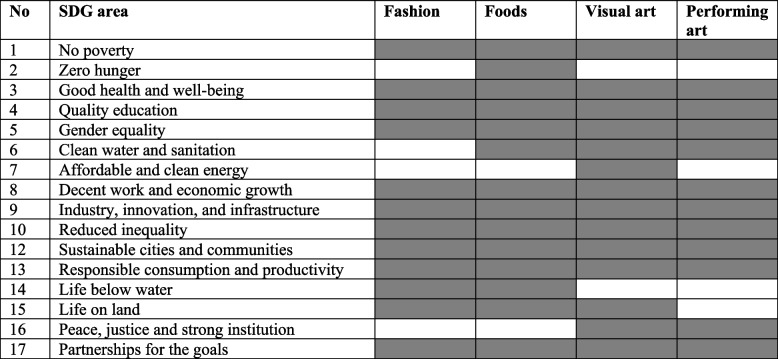
Creative economies and sustainable development goals

The fashion and food industries contribute to creating sustainable cities by adopting sustainable production and consumption practices. These movements include reducing waste, minimising water and energy consumption, using eco-friendly materials, and promoting circular economy principles. Collaborating with local communities, investing in skill development programs, and fostering inclusive supply chains help reduce economic and social inequalities. The survey found some social enterprises empower women in leadership roles and advocate for gender-responsive policies to help advance gender equality within their operations and throughout the supply chain.

Participants state that the creative community supports early-stage visual and performing-art entrepreneurs by offering physical workspace, mentorship, network access, and training programs. Many visual artists also work as art teachers or educators, sharing their knowledge and skills with others. The community supports local artisans and craftspeople, contributing to cultural vitality and economic sustainability in urban areas. They help entrepreneurs refine their business models, connect with potential investors, and accelerate their growth.

### Findings

*We found that social enterprises (1) encourage creative workers to embrace their cultural identities by providing a digital platform,*

Creating aesthetic products, from simple drawings to digital art, allows one to explore oneself and find hidden potential. However, many creative workers need more time to earn a steady income by accomplishing education or improving creative skills to meet the market. Only a few creative workers were born with destined talents and could establish an impressive career and a reliable income in the creative world. Hence, social enterprises help creative workers concern more about their passion than about financial benefits.

Embrace Cultural Diversity:

“We create a digital platform that celebrates and promotes cultural diversity.”

“We provide a space for creative workers from various cultural backgrounds to showcase their talents.”

“Our platform feature artists, designers, artisans, and performers who incorporate their cultural heritage into their work, fostering an environment that values and embraces different cultural identities.”

“Our digital platform empowers creative workers by giving them control over their artistic expression and allowing them to tell their own stories.”

Facilitating collaboration and networking:

“Our digital platform facilitates collaboration and networking among creative workers from diverse cultural backgrounds.

“Our organisation brings together artists, designers, and cultural practitioners who share similar cultural identities or have a common interest in exploring cultural themes.

“Our community allows creative workers to present their work in a way that reflects their cultural heritage, traditions, and values.”

“Our organisation fosters collaborative partnerships between creative workers and other businesses or organizations within their social networks.”

“Our organisation promotes partnerships that lead to joint marketing initiatives, co-branding opportunities, or collaborations on projects that amplify the visibility and reach of creative workers.”

“By tapping into their social networks, our firm facilitates the partnerships, creating mutually beneficial opportunities for creative workers and their customers.”

“Creative enterprises create communities and forums within their social networks where creative workers connect with their peers.”

“By providing a supportive environment, social enterprises enable creative workers to learn from each other’s experiences and gain valuable insights to enhance their customer engagement skills.”

*(2) Social enterprises support the creative workers in dealing with customers by leveraging their resourceful social networks,*

Social enterprises continuously make a living through their creative works by putting in much effort and resources to keep up with potential customers and gain stakeholder support. Creativity is inherent to the very nature of art, which is necessary to bring creative work to life in a restricted space, whether on canvas or online media. When the communities attempt to adapt to the dynamic business environment by bringing their creative work online, the young generation demonstrates a distinct capability to exciting levels for multimedia art. They reveal how self-confidence and a stoic work ethic to seize potential and generate opportunities. The creative works explore knowledge resources to work around online showcases, galleries, and other establishments to display their creative work. Other creative workers became content creators, taught after-school programs, or approached donors for financial support.

Access to global markets:

“Our digital platform provides creative workers access to global markets and audiences, enabling them to reach a broader customer base. This exposure encourages creative workers to embrace their cultural identities and create products or services rooted in their cultural heritage.”

“By facilitating market access, our platform supports economic empowerment and sustainability for creative workers.”

“These platforms allow creative workers to share experiences, exchange knowledge, and seek advice on customer interactions.”

Customer engagement

“Our organisation uses social networks to connect creative workers with potential customers, retailers, distributors, and other industry stakeholders.”

“Our firm helps creative workers gain visibility, expand their customer base, and access new market opportunities by leveraging the social networks.”

“Our firm facilitates customer engagement and feedback to the creative works by leveraging their social networks.”

“The creative works encourage customers to provide feedback, reviews, and testimonials about the creative workers’ products or services.”

“We collect feedback from customers to help creative workers understand customer preferences, make improvements, and build strong relationships with their customer base.”

Access to business resources:

“Creative enterprises leverage their social networks to provide creative workers with access to business resources that assist in dealing with customers effectively. These resources may include business tools, templates, guidelines, or workshops on topics such as pricing, contracts, customer relationship management, and sales techniques.”

“Creative enterprises urate these resources, making them easily accessible to creative workers by leveraging their networks.”

“By exploiting their resourceful social networks, creative enterprises provide a range of support to creative workers in dealing with customers. These efforts contribute to the sustainable growth of creative businesses, enhance customer relationships, and ultimately, create economic opportunities for creative workers.”

*(3) Social enterprises promote inclusive community governance by adopting a crowdfunding platform*

This study finds that some creative workers established community-based enterprises while others prefer to form an informal creative community network with a vision statement to support sustainable cities. The performance and strong networks allow them to access financial access from digital crowdfunding. The community leaders who owned the art gallery demonstrated total commitment to their artists. Showing something in their gallery means they are ready to support the creative works since only some people love and come to buy all the time. Some manage a freelance platform to build a network, from art professionals to freelance business opportunities. They also work a good relationship with donors, who typically have two distinct operations. The first is by invitation only, while another process is commercial.

Peer Support and Learning

Creative enterprises create communities and forums within their social networks where creative workers connect with their peers.

These platforms allow creative workers to share experiences, exchange knowledge, and seek advice on customer interactions.

By providing a supportive environment, social enterprises enable creative workers to learn from each other’s experiences and gain valuable insights to enhance their customer engagement skills.

Inter organisation learning.

Creative enterprises offer training and development programs specifically designed to help creative workers improve their customer engagement abilities.

Our programs focus on areas such as customer service, communication, negotiation, and marketing strategies.

Our business connects creative workers with industry experts, mentors, or experienced professionals who provide guidance and support by leveraging social networks.

Building a network of supporters

Excellent pilot projects often create a network of supporters who are passionate about the creative worker’s work and eager to see their future projects succeed.

By actively engaging with these supporters and creating opportunities for involvement, creative workers cultivate a network of individuals who may become long-term donors or advocates.

The social network become instrumental in spreading the word about the creative worker’s projects and attracting further support.

*(4) Social enterprises help the creative workers gain support from donors by generating some excellent pilot projects*

Some social enterprises respond to various discrimination and violence by bringing creative workers and social activists together to explore multiple mediums of expression to deal with social conflicts. In addition, some projects involve international non-profit organisations that focus on conflict prevention and resolution. The international donors proactively support the creative works to promote international partnerships in digital culture. Donors conducted various activities to help energise the creative workers by facilitating them to continue their creative activities through online exhibitions, workshops, and training activities. With support from community members, some market leaders established an internet marketplace, while others partnered with the government to conduct art education at local schools.

Demonstrating Proof of Concept:

“A well-executed pilot project serves as tangible evidence of the creative worker’s capabilities and the viability of their ideas.”

“By successfully demonstrating the feasibility and effectiveness of their work, creative workers build trust and credibility with potential donors.”

“The pilot project acts as a tangible representation of their vision and shows donors the potential for positive outcomes.”

Showcasing Innovation and Creativity:

“Pilot projects often involve innovative approaches, unique concepts, or creative solutions to existing challenges.”

“When executed excellently, these projects stand out and capture the attention of donors who are looking to support innovative ideas within the creative sector.”

“By showcasing their ability to think outside the box and bring fresh perspectives to their work, creative workers attract donors who are passionate about supporting creativity and artistic innovation.”

Engaging Stakeholders and Building Relationships

“Pilot projects often involve collaboration with various stakeholders, such as community members, organizations, or local institutions.”

“Creative workers engage stakeholders who may become potential donors or advocates for their work.”

“By involving stakeholders in the pilot project and building positive relationships, creative workers increase their chances of gaining support from donors who are connected to or aligned with those stakeholders.”

## Discussion

### Theoretical implication

This study extends the concept of entrepreneurial orientation by examining the practices in the Indonesian creative industry. The article provides insightful findings to the stewardship and stakeholder theories to understand how the creative enterprise achieves the SDGs. The first concern comes from the main idea of social enterprise that creative workers provide decent work by providing aesthetic products and helping creative workers generate a new identity. Identity also gives people access to social networks that provide support and shared values and aspirations. The findings confirm the identity theory and stewardship theory, which explains that initiative to accomplish social and environment emanates from a function of stewardship identity (Landon et al. 2021).

Thirdly, the stakeholder theory helps understand how creative workers adopt democratic governance, a critical element of a social enterprise model. The partnership with stakeholders comes in various types, following the interest and well-being of multiple stakeholders who support the organisational mission. The relationship with stakeholders can spring from market-based interest, voluntary activities, or other public contributions. In developing countries, economic activities with social missions informally adopt a cooperative way (Nyssens 2006; Defourny et al. 2020).

Another criterion of the social enterprise is participatory governance. The findings indicate that adopting social enterprise restrains business leaders from focusing on profit orientation by fabricating cultural identity. Knowledge exchange among creative workers has played a crucial role in preserving creative skills and traditions, which has evolved social changes within the cultural context and creative industry (Dias et al. 2020). This form of community transformation arises from projects designed to bring a community together to create a new identity (see Table 3).

The role of stakeholders is essential to promote a new identity that supports the 2030 Agenda for Sustainable Development. Stakeholder engagement in sustainability represents a powerful driver for value creation. For example, international development agencies designed funding mechanisms, including a community-based competition, to support public art initiatives addressing a locality’s pressing social problems. Therefore, the creative industry promotes identity by engaging its stakeholders through adoption, co-creation, and exploitation (Pucci et al. 2020).

Cultural identity contributes to well-being in which a particular culture provides feelings of security (McQuilten et al. 2020). The concept of stewardship culture continuously grows with collective orientation and mutual trust by promoting prosocial behaviour to manage community members from impulsive behaviour to protect the collective identity (Bormann et al. 2021). Hence, the identity theory explains that initiative to accomplish social and environment emanates from a function of stewardship identity (Landon, et al. 2021).

### Policy implication

Cultural diversity plays a vital role in promoting sustainable development. Policymakers should facilitate a learning process from different cultural contexts. Adopting a social enterprise model leads creative workers to work with mission-driven projects focusing on the targeted beneficiaries. This approach calls for coordination to address the barrier to participation in supply-side factors to drive higher engagement from the education policy system.

Secondly, cultural diversity also plays a pivotal role in job creation by enhancing competitive advantage by addressing social and ecological problems. Furthermore, supporting the social enterprise model encourages creative workers to work with mission-related activities in which they deliver creative works for a broader market than the targeted beneficiaries. Finally, enhancing the capacity of the creative communities is essential to support global citizenship to get involved in supporting cultural diversity by inviting intermediaries, such as curators, insurance, and other enablers.

It is also essential to help the creative community to adopt accountable reports by measuring their social and environmental impacts. This approach will justify how these creative workers improve continuously to achieve their social and environmental mission. In contrast, while commercial enterprises can measure their social and ecological impacts, such as through corporate social responsibility programs, this is secondary to their objective to make profits and could be deferred, if not jettisoned, if the social program contradicts their profit objectives.

In embracing these three elements, government recognition can vary from a formal definition of social entrepreneurship to appropriate incentives. Creative community leaders tend to adopt a business model driven by capital interest. Along with support from the community, the social and business mission mix logic with social entrepreneurship to gain financial sustainability (Defourny et al. 2020).

### Research limitation and future research

This study adopts interpretive analysis to understand the phenomena. This data collection began with offline focus discussion, observation, and interviews. However, the surveyors turned to online approaches following the restriction policy. The online comment attempted to harness the rich potential of qualitative data comes with some limitations, such as the number of research participants tend to be lower than the offline. Another issue is that online observation could have provided data as rich as offline observation, especially during the restriction policy. Future studies should examine other online business practices in the creative industry (Table 4).

**Table 4:** Coding analysis

**Open coding**	**Axial coding**	**Selective coding**
A digital platform A space for creative workers Awareness Cultural heritage Cultural identities Diverse cultural backgrounds Engaging with a broader audience Promotes cultural diversity Reflects their cultural heritage Showcase their talents	1.1. Embrace cultural diversity:	1. Social enterprises play a significant role in encouraging creative workers to embrace their cultural identities by providing a digital platform
Co-branding opportunities, or collaborations on projects Creative workers and their customers Facilitates the partnerships Joint marketing initiatives, Mutually beneficial opportunities Social networks Tapping into social networks Reach of creative workers	1.2. Collaborative partnerships:	
Access to global markets A broader customer base Economic empowerment Facilitating market access Sustainability for creative workers	2.1. Access to global markets:	2. Social enterprises support creative workers in dealing with customers by leveraging their resourceful social networks
Customer engagement Customer reviews Customer testimonials Customer preferences Encourage customers to provide feedback Leveraging their social networks Relationships with their customer base	2.2. Customer engagement and feedback	
A range of support Access to business resources Business tools, templates, and guidelines Create economic opportunities Dealing with customers effectively Easily accessible to creative workers Enhance customer relationships Pricing, contracts, customer relationship Leveraging their networks, Resourceful social networks Sustainable growth	2.3. Access to business resources:	
Communities within their social networks connect with their peers Customer engagement skills Enable creative workers to learn Exchange knowledge Gain valuable insights Seek advice on customer interactions Share experiences	3.1. Peer support and learning	Social enterprises promote inclusive community governance by adopting a crowdfunding platform
Customer engagement abilities Customer service, Communication, negotiation, Experienced professionals Industry experts Leveraging social networks Marketing strategies Training and development programs	3.2. Inter organisational learning	
A network of individuals A network of supporters Passionate about the creative works Future projects succeed Engaging with supporters Long-term donors or advocates Social network Spreading Attracting further support	3.4. Building a network of supporters	
A tangible representation A well-executed pilot project Credibility with potential donors Tangible evidence Viability of their ideas Feasibility and effectiveness of their work	4.1. Demonstrating proof of concept	4. Excellent pilot projects play a crucial role in helping creative workers gain support from donors by showcasing their talent, vision, and potential impact
Artistic innovation Attention of donors Creative solutions Existing challenges Fresh perspectives Innovative approaches Relevant data Showcasing Unique concepts	4.2. Showcasing innovation and creativity	
Collaboration with various stakeholders Potential donors Stakeholders in the pilot project Positive relationships, Chances of gaining support Aligned donors	43. Engaging stakeholders and building relationships	

The second limitation comes from the number of participants from 12 cities in Indonesia. Observing participants in 12 cities provides rich data, which implies an overload of information. That may become an advantage, but there are also disadvantages, such as bias or irrelevant in explaining the phenomena. This experience aligns with the categorisation theory that the researchers initially seek to elaborate the categorisation structures on exploring strange behaviours, which leads to another research topic. Hence, future studies are encouraged to develop the categorisation into factor analysis to develop an empirical approach.

## Conclusion

This article examines how social enterprises support the creative industry in achieving sustainable development. The research design extends the main research question into sub-research questions followed by open-ended questions to understand the phenomena. The initiative to support the SDG aligns with their intention to create cultural identity. Hence, business leaders, donors, investors, and policymakers help young creative people to realise the nature of their choice to create a new identity. Therefore, this study extends the concept of social enterprise by exploring creative works which bring some unique elements to support sustainable development goals. The social enterprise context also helps the analysis to extend the stakeholder theory by highlighting the role of creative workers as beneficiaries, the community leaders as the primary business enablers, and the broader community as a market. Finally, the results also contribute to the stewardship theory by highlighting the role of cultural identity in shaping the social enterprise model.

## Data Availability

Data is available at British Council Indonesia Youtube Link: https://www.youtube.com/@BritishCouncilIndonesia/videos.
